# A study on the mechanical strength of alfalfa seeds stimulated with continuous laser light

**DOI:** 10.1038/s41598-024-81725-z

**Published:** 2024-12-03

**Authors:** Agata Dziwulska-Hunek, Mariusz Szymanek, Arkadiusz Matwijczuk, Norbert Leszczyński

**Affiliations:** 1 Department of Biophysics, University of Life Sciences in Lublin, Akademicka 13, 20-950 Lublin, Poland; 2 Department of Agricultural, Forest and Transport Machinery, University of Life Sciences in Lublin, Głęboka 28, 20-612 Lublin, Poland

**Keywords:** Plant sciences, Ecology

## Abstract

The presented study explored the impact of He-Ne laser light stimulation on basic, selected mechanical properties of alfalfa seeds. The following parameters were considered: destructive force (F), destructive force work (W), and absolute longitudinal deformation (dL). Moreover, the single seed mass (m) was measured prior to the compression test, and the ratios of destructive force to absolute longitudinal deformation (F/dL) and destructive force work to single seed mass (W/m) were calculated. Subsequently, the mass of 1000 seeds was determined using RADWAG XA110.3Y scales, and the geometric dimensions of seeds were measured using a Keyence VHX-2000 microscope. Prior to the compression test, the seeds were subjected to laser light stimulation (λ = 632.8 nm, surface force density = 6 mW·cm^− 2^) with the exposure time of, respectively: 0 s (control – no stimulation – C), 30 s (L30s), 1 min (L1), 2 min (L2), and 3 min (L3). We observed an increase in most of the mechanical parameters measured under the influence of all the laser light stimulation variants. The only exception was the F/dL ratio, which decreased after 2 and 3-minute exposure, as compared to the control. The results evidenced increased mechanical strength of the stimulated seeds, which may prove significant to the cultivation of new, more resilient cultivars.

**Supplementary Information:**

The online version contains supplementary material available at 10.1038/s41598-024-81725-z.

## Introduction

Alfalfa (*Medicago sativa* L.), known as ‘the queen of fodder plants,’ has long been one of the most common fodder crops in the world^1–5^. It is usually given to animals in the form of green fodder, hay, or haylage, as well as in processed forms such as protein-xanthophyll concentrate (PX) or leaf extract (EFL)^6,7^. Moreover, alfalfa seeds are used in the food industry as a source of edible sprouts for sandwiches and salads^8–10^. The major problem with these plants is the presence of hard seeds in the seed material, which can impact germination rates and profitability of sprout production^11^. This is due to the specific structure of the seed coat that determines the plant’s industrial viability and is primarily influenced by environmental and genetic factors^12,13^. In some seeds, temporary inhibition of germination can be observed due to their inability to absorb water through the coat, which results in lower moisture content. In the context of alfalfa, seeds with this type of coat structure are classified as hard seeds^14,15^. Germination is affected by several environmental factors, such as availability of light, temperature, and humidity, but also the conditions of seed ageing and storage^16,17^. In the case of plants with small seeds, such as alfalfa, lettuce, or tomato, light serves as the primary regulator of germination^18^, although many species are able to germinate even in its absence^13^.

The value of plant seeds is determined by their germination rate, yield structure, and ultimate fodder quality. Therefore, in the context of commodity crops, it is important that the seeds selected for sowing are of the highest possible grade. To ensure this, seeds are refined using a variety of processing methods, including chemical and mechanical (dressing, growth regulators, etc.) as well as physical (laser light, magnetic field, etc.)^19,20^. Physical methods are generally considered more environmentally friendly. Numerous studies conducted in this context have focused on the germination rates and yields of various arable crops and vegetables. Their results revealed tangible benefits of employing electromagnetic stimulation^20,21^.

Literature reports mention that a light dose with the surface force density of 6 mW·cm^− 2^ and the 3- and 5-fold exposition to He-Ne laser light resulted in a reduction in the amount of Alternaria fungi isolated from alfalfa seed material^22^.

The use of laser light stimulation has been increasingly popular in agriculture in recent years. It has been shown to improve the growth and development of crops, as well as benefit the natural environment by reducing the need for chemical fertilizers. The mechanism of laser light stimulation involves conversion of light energy into chemical energy, which activates certain physiological and biochemical processes within seeds. This directly influences the dynamics of plant growth. Seeds have the capacity to absorb and store radiative energy^23,24^. The phytochrome is a photoreceptor reacting to the presence or absence of light, composed of a protein part and a chromophore. It is activated by red light. Hence, it plays a key role in the germination of seeds particularly susceptible to light^25^. Stimulation is most often achieved at a wavelength of 632.8 nm, emitting red laser light absorbed in the phytochrome^21,26^. However, despite numerous studies on the topic, the exact mechanism of laser light stimulation is yet to be determined. It has been observed that it elevates the bioenergetic potential of seeds, leading to increased phytochrome activity, which triggers physiological and biochemical processes in the seeds. During germination, enzymatic activity related to phytochrome increases entropy and internal energy levels^27^. The effect of stimulation with 632.8 nm laser light activates the phytochrome in the seed, which changes the balance of growth regulators and has a beneficial effect by interrupting the seed dormancy period in the process of germination^28^.

Seeds are likely to sustain mechanical damage during harvest and de-chaffing processes, and such damage makes them more vulnerable to pathogenic infections and germination abnormalities^15^. Susceptibility to such damage is influenced by the structure and mechanical strength of the seed coat, as well as the relevant moisture levels^29^. The cultivation of annual and perennial legumes can be significantly hindered by the physical condition of seeds after storage, which in turn is largely dependent on their physiological structure. If the seed coat is impenetrable to water, germination may be arrested even for several years^30^.

The research problem analyzed in this paper pertains specifically to the mechanical properties of alfalfa seeds after (additional) stimulation with He-Ne laser light. Alfalfa sowing material tends to be notoriously hard seeded. Reports available in the literature mention various proportions of hard seeds in alfalfa seed material, typically ranging from 22 to 37%, but sometimes as high as 50%^15,31^. This directly translates to lower germination capacity. Hard seeds do not germinate or rot even under favorable conditions: suitable temperature, moisture, aeration, and insolation. The share of hard seeds is common to many legumes, and obviously, the resulting longevity of seeds over extended dormancy periods is a way of ensuring the species’ long-term survival. This is exactly what allows the seeds of some species to germinate even after a 150 or 200-year vegetation period. In the context of the above as well as the observed seed response to light and the reported positive impact of red light on the process of seed germination, particularly in the case of very heliophilous alfalfa, we decided to explore this topic in greater depth and from a variety of angles. Notably, this particular problem has only very rarely been considered in scientific literature to date. Meanwhile, it is well known that seeds are subject to various types of damage during harvest, transport, storage, and processing, which can lead to qualitative as well as quantitative losses. Information on the mechanical characteristics of seeds can also facilitate the design of new, improved agricultural and horticultural machinery and implements. Furthermore, such information can also prove useful to efforts aimed at developing new alfalfa cultivars.

The presented study aimed to determine the impact of He-Ne laser light stimulation on selected mechanical characteristics of alfalfa seeds. To this end, parameters such as the destructive force (F), destructive force work (W), and absolute longitudinal deformation were measured. Furthermore, the single seed mass was measured prior to the compression test, as were the ratios of destructive force to deformation (F/dL) and destructive force work to single seed mass (W/m). Additionally, the mass of 1000 seeds and the geometric dimensions of seeds were determined. Additionally, we determined the germination capacity and share of hard seeds. One-way ANOVA analysis was conducted for the relevant variables, after which the zero hypothesis H_0_ was formulated as to whether the following parameters: single seed mass, destructive force, work of destructive force, absolute longitudinal deformation at destructive force, contractive compressive strength index, and destructive force work to seed mass ratio were affected by laser light stimulation. Furthermore, we aimed to determine whether seed treatment with laser light influenced the germination capacity and share of hard seeds.

## Materials and methods

**Fig. 1:**
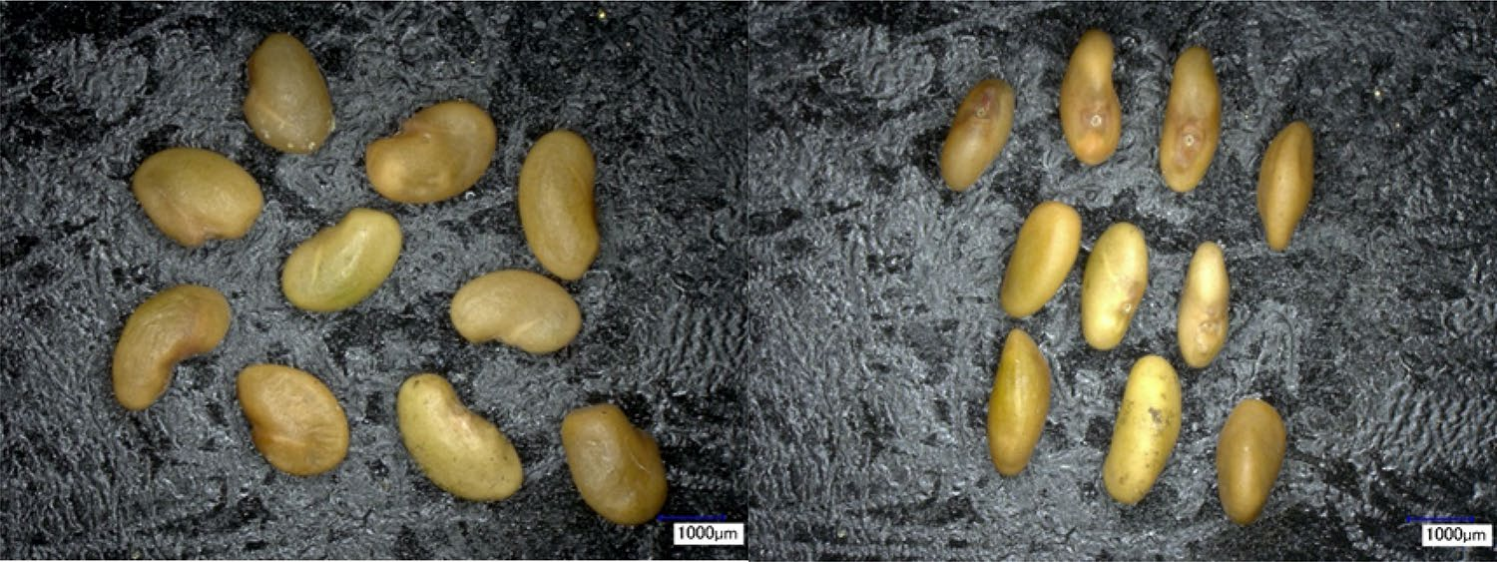
Microscopic images of alfalfa seeds in positions a and b (Table 1). Photo by Norbert Leszczyński.

**Fig. 2:**
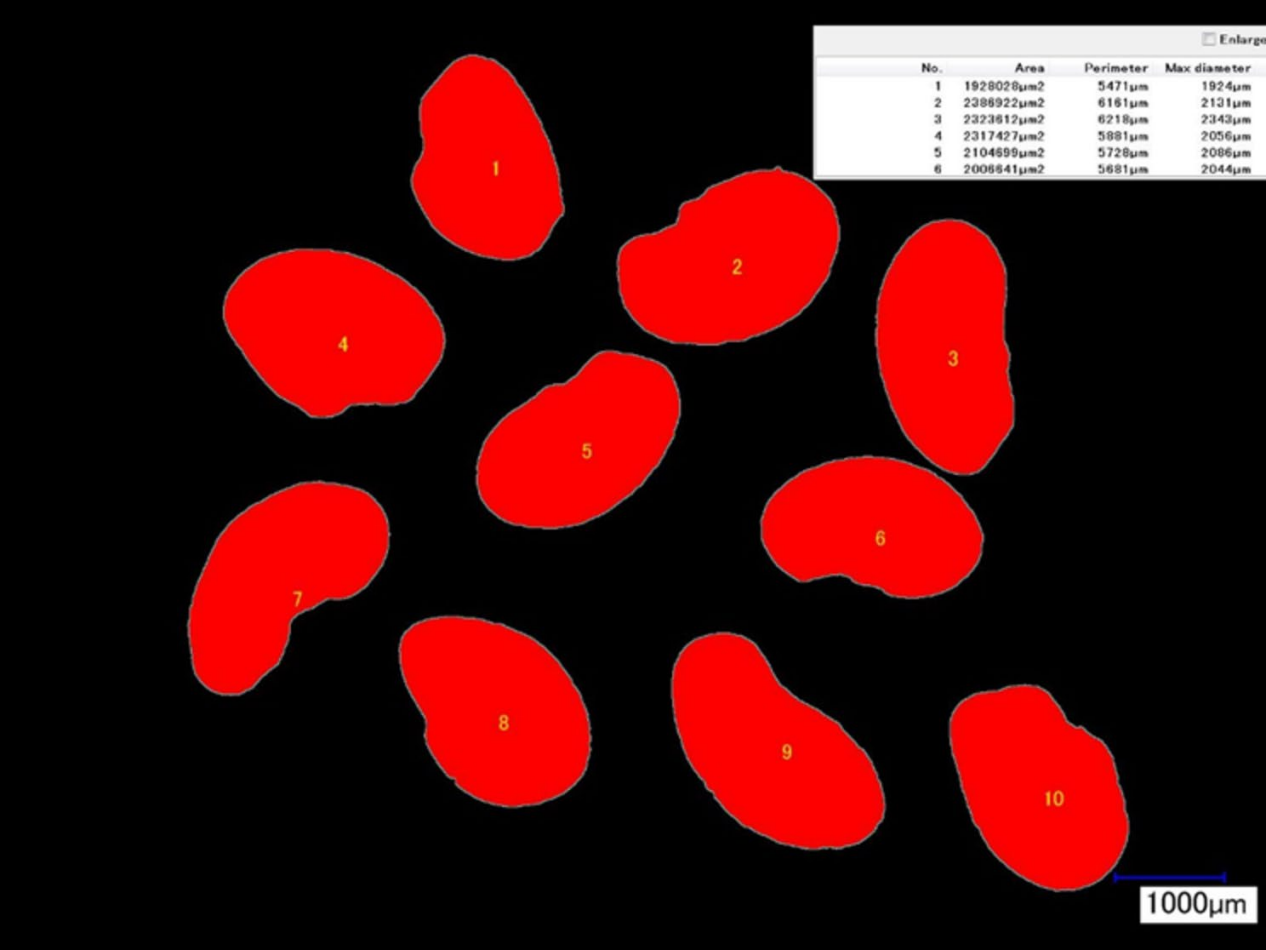
Photograph taken during the measurement of seed dimensions. Photo by Norbert Leszczyński.

The research material comprised four-year-old seeds of ‘Ulstar’ alfalfa with the recorded germination rate of 84% in the year of the harvest (2013)^32–34^. The seeds could be described as small, kidney-shaped, yellow and brown in color. Table 1 presents relevant characteristics of the studied alfalfa seeds in terms of the mass of 1000 seeds and their geometric dimensions. The mass of 1000 seeds was determined using a RADWAG XA 110.3Y scale accurate to 1 mg. Furthermore, the geometric dimensions of the seeds were measured using a Keyence VHX-2000 digital microscope (20x magnification). Measurements were taken in two seed positions (Fig. 1). Figure 2 presents images of 10 seeds taken for the purposes of microscopic interpretation of the obtained measurement results.

**Table 1:** Characteristics of Ulstar alfalfa seeds.

Physical qualities	Replication			Mean
	1	2	3	
Mass of 1000 seeds (g)	1.729 ± 0.027	1.712 ± 0.028	1.726 ± 0.032	1.722
Geometric dimensions	Position a			
Area (µm^2^)	1,804,201 ± 212,876	2,273,944 ± 257,889	2,137,675 ± 207,148	2,071,940
Perimeter (µm)	5668 ± 332	6120 ± 444	5800 ± 340	5863
Max diameter (µm)	2061 ± 104	2232 ± 170	2064 ± 200	2119
Min diameter (µm)	1161 ± 79	1324 ± 86	1356 ± 101	1280
Geometric dimensions	Position b			
Area (µm^2^)	1,293,400 ± 131,430	1,543,826 ± 176,315	1,533,188 ± 142,330	1,456,805
Perimeter (µm)	5257 ± 306	5468 ± 367	5356 ± 400	5360
Max diameter (µm)	2054 ± 134	2216 ± 170	2056 ± 199	2109
Min diameter (µm)	831 ± 34	898 ± 53	964 ± 82	928

The mass of 1000 seeds and seed dimensions are provided for 10 randomly selected seeds measured in triplicate. ± standard deviation

**Fig. 3:**
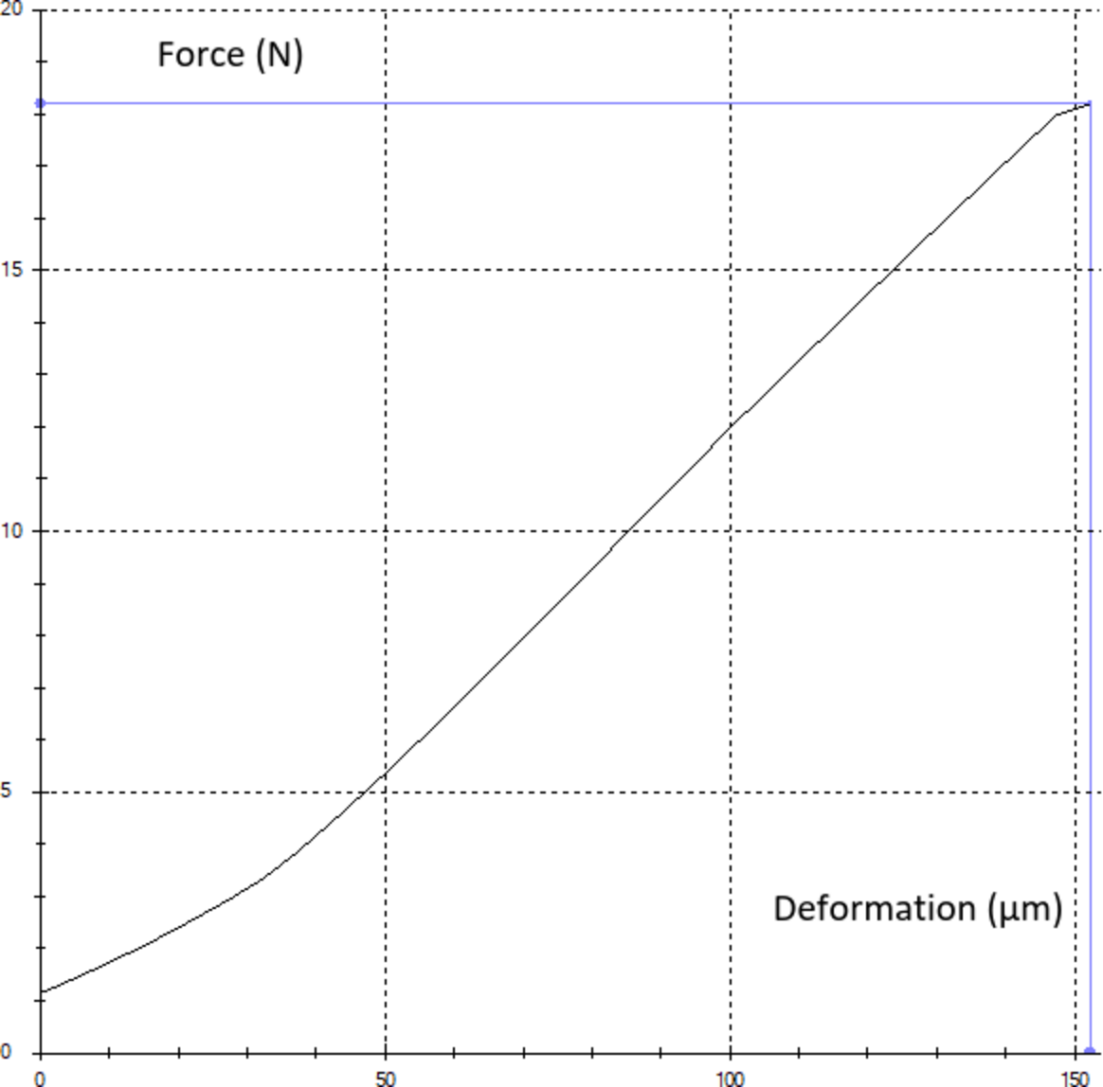
Example single seed compression curve for force and deformation.

We assessed the compressive strength of seeds after laser He-Ne light stimulation (wavelength = 632.8 nm, surface force density = 6 mW·cm^− 2^) applied as follows: control – C (no stimulation): 0 s, stimulation with laser light with the exposure time of, respectively: 30 s (L30s), 1 min (L1), 2 min (L2), and 3 min (L3). Laser stimulation was applied using a device of our own design^35^, with a vertical beam of red light reaching the seeds arranged in a single layer inside a dish. The moisture content of the tested seeds was 6%, measured with the weighing and drying method using a RADWAG Max50/1WH scale. The mechanical tests were conducted at the Department of Agricultural Forestry and Transport Machines. The compression test was performed using a Zwick/Roell Z005 machine (head accuracy 1%, or 0.5 N) and Test Xpert II.V3.5 data recording software by Zwick. The compressive strength of individual seeds was measured between two parallel surfaces. A measuring head with the maximum pressing force of 50 N was used. The measurement was carried out until the seed burst, recording changes in the loading force as a function of the displacement of the measuring head. The machine automatically recorded the force destroying the seed, i.e. the maximum value of the force, followed by a sudden drop in force caused by the seed splitting. The compression speed was 3 mm per 1 min. 30 seeds selected at random from each batch of seed material were used in the test. The tests were conducted at 20 °C ± 1 °C. The seeds were compressed perpendicularly to the cotyledon surface at the constant speed. Prior to the compression, the single seed mass was measured using a RADWAG XA 110.3Y electronic scale accurate to 1 mg. Figure 3 presents an example seed compression curve for force and deformation.

The measured parameters were: single seed mass – m, destructive force – F, Work of destructive force – W, absolute longitudinal deformation at destructive force – dL, contractive compressive strength index – F/dL, destructive force work to seed mass ratio W/m.

We additionally measured germination capacity and share of hard seeds. The germination process took place in a climate chamber under constant temperature (21 °C ± 1 °C), illumination 12 h/12 h (5 warm fluorescent lamps with the total flux of 6,000 lumens), on Petri dishes of 90 mm in diameter and 15 mm in depth covered with three layers of filter paper. Next, the paper was moistened with 6mL of distilled water per dish.

The germination capacity and share of hard seeds were measured after 10 days. Seeds showing germination capacity were defined as those with two seed leaves and a root. Hard seeds were those that did not germinate in the designated period and were characteristically hard.

Prior to sowing, seeds were stimulated with He-Ne laser light (wavelength = 632.8 nm, surface force density = 6 mW·cm^− 2^) over 0 s (control), 30 s, 1 min, 2 min, and 3 min. Next, they were sown onto the Petri dishes, 100 seeds per dish. Each stimulation variant was tested in triplicate.

### Statistical analysis

The obtained results were processed statistically using STATSTICA 13.0 software. Statistical differences between the respective study groups were subjected to the ANOVA analysis, NIR test with the significance threshold of *p* = 0.05. The relations between variables were determined using Pearson’s linear correlation coefficient.

## Results and discussion

Table 2 presents mean values of the mechanical strength parameters measured for alfalfa seeds. The results of one-way analysis show the mean values of the impact of laser light stimulation on the respective mechanical parameters of the seeds (Table S1). The single seed mass increased under the influence of laser light stimulation and was, respectively: 2.6% (L2), 5.0% (L3), and 5.3% (L1) higher relative to the control (C), although the differences were not statistically significant (*p* > 0.05). The seed phytochrome absorbed red light, increasing the seed’s energetic potential^27,28^. He-Ne laser light activated various biological reactions in the seeds, triggering biochemical and physiological processes that may have also influenced seed mass.

**Table 2:** The values of strength parameters of an alfalfa seed.

Laser light stimulation	m (mg)	F (*N*)	W (*N* · mm)	dL (mm)	F/dL (*N* · mm^− 1^)	W/m (*N* · mm · mg^− 1^)
C	1.700^ab^ ± 0.298	16.13^a^ ± 2.65	1.532^b^ ± 0.329	0.164^b^ ± 0.017	98.59^a^ ± 13.06	0.906^b^ ± 0.149
L30s	1.613^b^ ± 0.274	17.35^a^ ± 3.25	1.725^a^ ± 0.410	0.171^ba^ ± 0.017	101.58^a^ ± 15.81	1.085^a^ ± 0.263
L1	1.790^a^ ± 0.253	17.51^a^ ± 2.31	1.749^a^ ± 0.357	0.173^a^ ± 0.019	101.32^a^ ± 10.08	0.976^b^ ± 0.158
L2	1.744^ab^ ± 0.233	16.88^a^ ± 2.55	1.706^ba^ ± 0.338	0.174^a^ ± 0.338	97.26^a^ ± 11.50	0.985^a^ ± 0.205
L3	1.785^a^ ± 0.362	17.03^a^ ± 3.27	1.680^ba^ ± 0.422	0.171^ba^ ± 0.017	99.63^a^ ± 14.55	0.956^b^ ± 0.224
Mean	1.726	16.98	1.679	0.170	99.66	0.982

C – control (seeds not stimulated with laser light): 0 s, L30s, L1, L2, L3 – He-Ne laser light stimulation with the exposure time of, respectively: 30 s, 1 min, 2 min, and 3 min. a–b – different letters in respective columns indicate statistical differences between the tested samples and the control (*p* = 0.05), *n* = 30. ± standard deviation.

The He-Ne laser light increased the destructive force measured for alfalfa seeds by 4.6% (L2) to 8.6% (L1) relative to the control. However, no statistically significant differences were observed (*p* > 0.05). The destructive force work increased for all laser light exposure times. An increase of 12.6 and 14.2% was observed for the 30 s and 1 min exposure times, respectively, and the differences were statistically significant (*p* < 0.05). Moreover, the ratio of destructive force resistance was higher, by between 1.1% and 3.0%, after stimulation at three different exposure times, but with no statistically significant differences observed (*p* > 0.05). The ratio of destructive force to the single seed mass increased for all stimulated samples relative to the control. Statistically significant differences (*p* > 0.05) were observed for 30 s and 2 min exposure times, with the ratio increasing by, respectively, 19.8% and 8.7%.

**Fig. 4:**
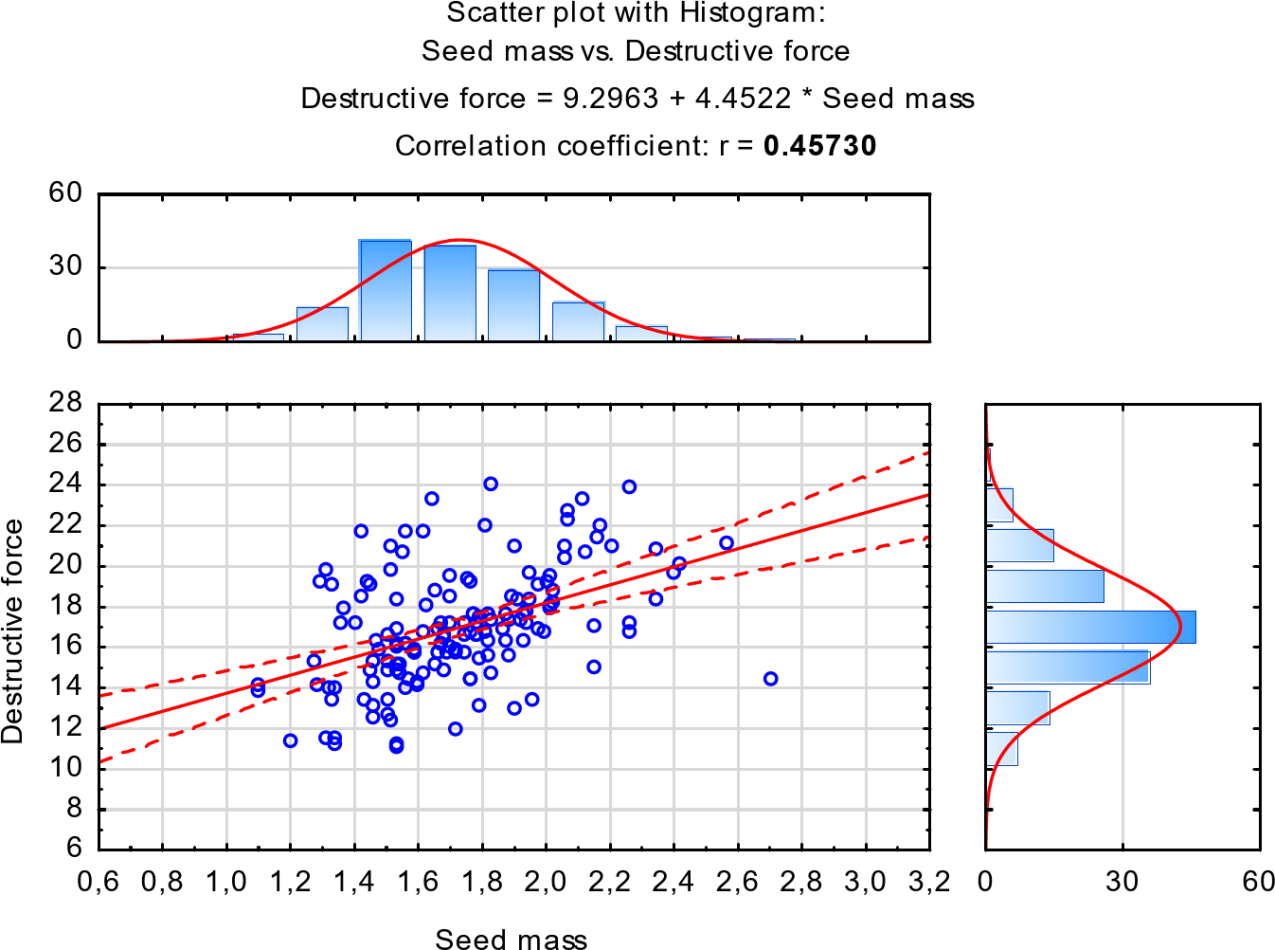
Scattering plot with histogram for the correlation between: destructive force and single seed mass.

**Fig. 5:**
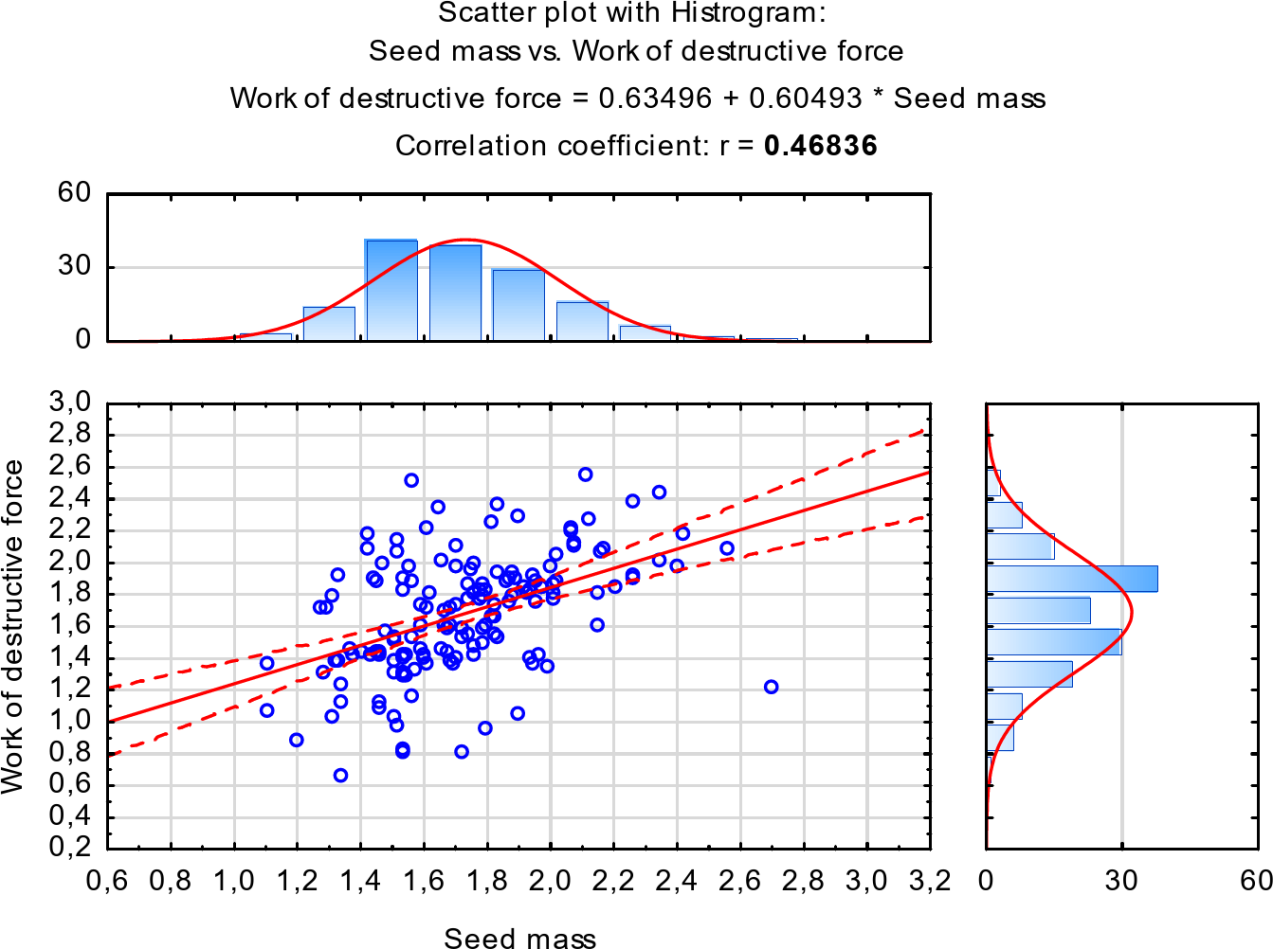
Scattering plot with a histogram for the correlation between: destructive force work and single seed mass.

The scattering plots show two linear correlations between the presented variables: destructive force and seed mass (Fig. 4), and destructive force work and single seed mass (Fig. 5). Positive values of the coefficients indicate the direction of the relations.

In the case of alfalfa from. the European region, the average mass of 1,000 seeds can vary, year to year, between 1.6 and 2.2 g in the year of sowing, respectively: 1.6–1.9 g and 1.8–1.9 g in the subsequent two years^36^. The average mass of 1,000 seeds measured in our experiment was 1.72 g.

To date, the available literature discussing the mechanical qualities of seeds has been predominantly focused on plants such as lupine, broad bean, soybean, pea, or lentil. The studies analyzed the mechanical strength of seeds depending on their mass, thickness, and moisture content. Reports have also been published with regard to the impact of laser light stimulation on germination, sprouting and yields, as well as the content of photosynthetic pigments, but there have been no studies focusing specifically on its influence on the mechanical strength of seeds^13,19–21^.

Kuźniar et al.^29^ analyzed the durability of legume seeds (lupine, broad bean, soybean) relative to their mass and thickness. The researchers observed that the work required to destroy the seed coat was the highest for narrowleaf lupine, while the highest destructive force was recorded for broad bean. The mass measured for broad bean (450.0–497.6 mg) was between 2 and 4.5 higher than that of yellow-leaf (110.6–135.7 mg) and narrow-leaf lupine (131.0–166.7 mg) or soybean (179.8 mg). The respective destructive forces were measured at between 158.6 N (soybean) and 634.7 N (broad bean). As clearly follows from the above, the destructive force was positively correlated with the size of the seed as with growing mass, all the analyzed leguminous seeds showed increased damage resistance, while increased thickness correlated with a reduced extent of deformation.

In a study conducted by Zhang and Zhao^37^, it was reported that the results of the compression tests conducted for three alfalfa cultivars depended on the shape and size of the seeds, with the destructive force registered at between 19 N and 30 N. In our research, the single seed mass was observed to increase under the influence of laser light stimulation with the exposure time of 1 min, 2 min, and 3 min, which seems to be the reason for elevated destructive force results compared to the control. In our case, the registered destructive force was considerably lower, reaching between 16.33 N and 17.51 N, which may have been due to the size of the alfalfa seeds tested. Moisture may have also contributed to the results, in the case of all seeds included in the presented study it was constant at 6%. The moisture level is dependent on the age of seeds (in our case the seeds were four years old), as literature indicates that it tends to decrease in longer storage. In some studies on the mechanical properties of seeds, e.g. by Żuk- Gołaszewska^12^ or Kuźniar et al.^29^, the tested seeds had a water content as high as 12% (red clover) or 13% (selected legumes).

As emphasized by Żabiński and Mudryka^38^ as well as Kuźniar et al.^39^, the moisture content of seeds has a significant impact on their mechanical characteristics. An increase in the moisture content of soybean from 7 to 19% resulted in a reduction of forces and elasticity modulus with a simultaneous increase in energy and deformation. At the same time, in a study by Rybiński et al.^40^ on the mechanical strength of seeds, the highest levels of destructive forces, deformation and energy for the moisture content under 10% were reported for various leguminous plants (lupine, pea, lentil).

The moment of seed ageing depends on the plant species, structure and composition of the seed, as well as the method of storage. This is why maintaining adequate temperature and moisture of seeds in storage is so vital^31^. Dry seeds tend to be harder and less flexible, which translates to higher destructive force parameters.

The results of our additional experiments concerning the impact of laser light stimulation on germination capacity and share of hard seeds are presented in Figs. S1 and S2. Germination capacity was recorded at 65–80%. We observed a considerable impact of laser light stimulation in this context with an increase of 10–14% relative to the control. The only exception was the L1 group, where a 7% decrease was registered. The share of hard seeds in the sowing material after the laser light treatment ranged between 19% (L30s) and 23% (L1). As follows from the statistical analysis, laser light stimulation resulted in significant differentiation of the mean germination capacity value at *p* < 0.05, while the share of hard seeds was not significantly affected (*p* > 0.05). As reported by Wilczek et al.^22^, stimulation with He-Ne laser light improved germination of alfalfa seeds, increasing the relevant levels by 2–3% relative to the control group. Seed germination was observed in 95–97.8% seeds and the share of hard seeds ranged between approx. 2 and 4%. In another study, stimulation of wheat seeds with dipole laser light (wavelength 630 nm, 20-minute exposure) improved germination was observed^23^. In a study exploring the effects of stimulating brinjal seeds with He-Ne laser light, a significant increase in terms of germination was reported^26^. In a study on the effects of laser light stimulation on sainfoin seeds (eight months after harvest), germination rates were observed to increase from 80 to 85%, while the share of hard seeds was reported at 2–5%^41^. The best germination results of 90% were reported for two cultivars: *Jacaranda mimosifolia* and *Prosopis laevigata* after 120s exposure to He-Ne light^42^. In turn, in our own study, exposure to laser light for 30s and 2 min yielded the best germination result of 80%.

The mechanical strength of seeds increased after laser light stimulation, which may be associated with germination capacity that tends do decease as seeds age, which ties to greater hard seed share. The analyzed seeds were four years old, and the compression test was conducted at a constant moisture content (6%). As reported in another study, alfalfa seeds stored for a period of 30 months showed 80% germination capacity at 6.4% moisture content, while the initial germination capacity had been 100%. In turn, the share of alfalfa hard seeds was between 10 and 22%^43^.

## Conclusions

To recapitulate our results regarding the impact of laser light on the mechanical characteristics of alfalfa seeds, it can be concluded that resistance to mechanical damage increased after the stimulation. Any type of mechanical damage can reduce the viability of agricultural material, particularly seeds intended for sowing or further processing. The destructive force and destructive force work parameters increased for all tested variants of laser light stimulation.

We observed a significant impact of laser light stimulation on the germination capacity, while the share of hard seeds was not affected. Seed germination is closely related to their moisture content which decreases over time in storage as the percentage share of hard seeds increases. The decreased germination capacity is due to seed dormancy dictated by seed hardness and water impermeability of the seed coat.

Moreover, it can be observed that the germination capacity of seeds tends to decrease in storage while their mechanical strength increases with the changing percentage of hard sees present. Naturally, the longer the storage period, the lower the overall moisture content of the seeds. Older seeds tend to show lower values of water content and lower germination capacity, as well as higher resistance to compression.

The presented results related to the mechanical properties of seeds can provide data useful for designers of harvest and storage equipment. They can also contribute to a better characterization of the particular plant species and the development of new, more resilient cultivars.

## Electronic supplementary material

Below is the link to the electronic supplementary material.

Supplementary Material 1

## Data Availability

Data will be available on request. Correspondence and requests for materials should be addressed to A.D.-H.
