# Supplementary material for: A study on the mechanical strength of alfalfa seeds stimulated with continuous laser light
Source: Sci Rep. 2024 Dec 3;14:30114. doi: 10.1038/s41598-024-81725-z (PMC11614908; doi:10.1038/s41598-024-81725-z)
Supplement: Supplementary file 1 — Supplementary Material 1 [file 41598_2024_81725_MOESM1_ESM.pdf]

Variable and source of variation	Laser light stimulation of seed		
	df	F	p
Single seed mass	4	1.933	0.108
Destructive force	4	1.079	0.369
Work of destructive force	4	1.578	0.183
Absolute longitudinal deformation at destructive force	4	1.554	0.190
Contractive compressive strength index	4	0.576	0.680
Destructive force work to seed mass ratio	4	3.052	0.019
Germination capacity	4	207.8	0.000
Share of hard seed	4	1.476	0.281
df – degrees of freedom, F –Fischer test, p – significance level p < 0.05			

Table S1. One-way ANOVA illustrating the impact of light laser stimulation on single seed mass, destructive force, work of destructive force, absolute longitudinal deformation at destructive force, contractive compressive strength index, destructive force work to seed mass ratio and germination capacity and share of hard seed.

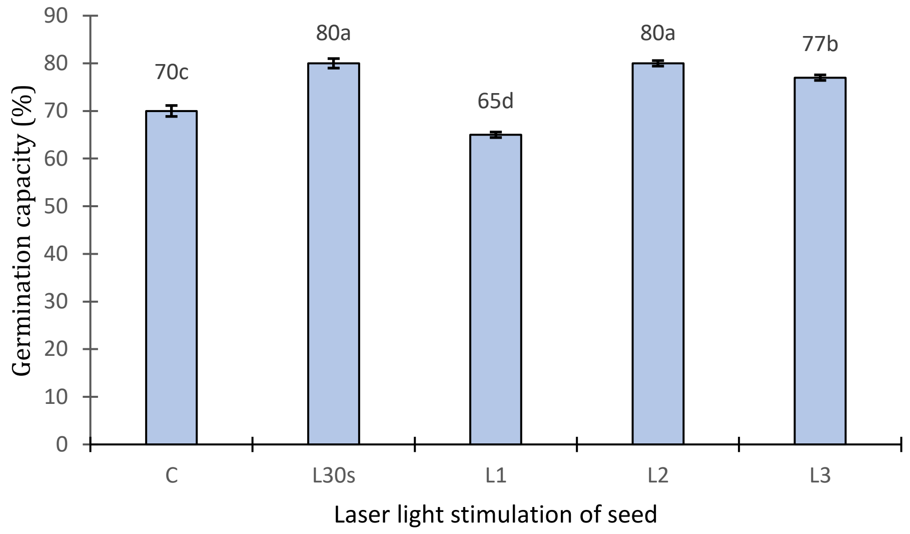

Figure S1. Effect of laser light stimulation on germination capacity of alfalfa seeds

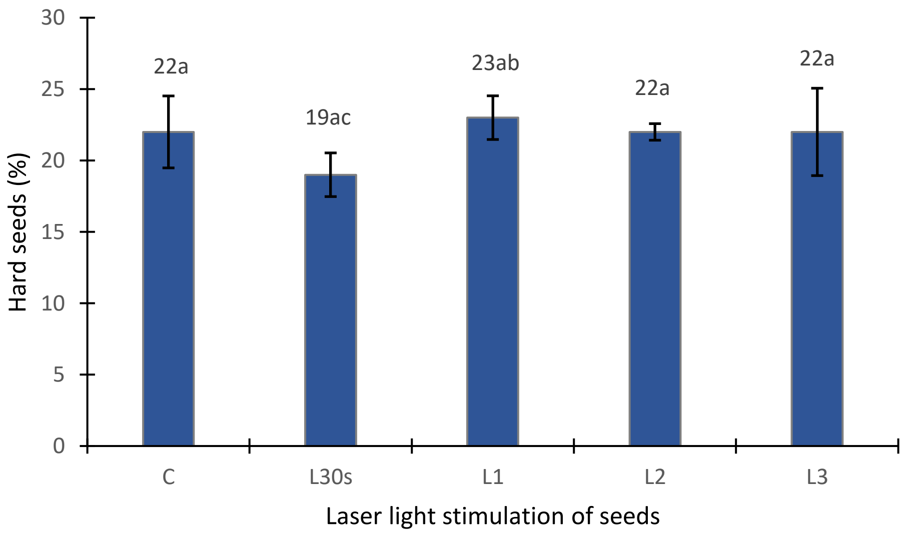

Figure S2. Effect of laser light stimulation on hard seeds in alfalfa
